# Supraphysiological Levels of IL-2 in Jak3-Deficient Mice Promote Strong Proliferative Responses of Adoptively Transferred Naive CD8^+^ T Cells

**DOI:** 10.3389/fimmu.2020.616898

**Published:** 2021-01-28

**Authors:** Gil-Woo Lee, Sung-Woo Lee, Juhee Kim, Young-Jun Ju, Hee-Ok Kim, Cheol-Heui Yun, Jae-Ho Cho

**Affiliations:** ^1^ Division of Integrative Biosciences and Biotechnology, Pohang University of Science and Technology, Pohang, South Korea; ^2^ Medical Research Center for Combinatorial Tumor Immunotherapy, Department of Microbiology and Immunology, Chonnam National University Medical School, Jeonnam, South Korea; ^3^ Immunotherapy Innovation Center, Chonnam National University Medical School, Hwasun Hospital, Jeonnam, South Korea; ^4^ Department of Agricultural Biotechnology and Research Institute of Agriculture and Life Sciences, Seoul National University, Seoul, South Korea

**Keywords:** interleukin-2, naive CD8^+^ T cells, antigen-independent proliferation, Janus kinase 3, activated CD4^+^ T cells, CD4^+^ regulatory T cells

## Abstract

The antigen-independent, strong proliferative responses of naive CD8^+^ T cells have been well demonstrated in a particular strain of mice lacking IL-2 receptors. This type of proliferation is mainly driven by common gamma-chain (γ_c_) cytokines, such as IL-2, IL-7, and IL-15, present at abnormally high levels in these mice. Similarly, in the present study, we showed that mice lacking Janus kinase 3 (Jak3), a tyrosine kinase crucial for γ_c_ cytokine signaling, could induce strong proliferation of adoptively transferred naive CD8^+^ T cells. This proliferation was also independent of antigenic stimulation, but heavily dependent on IL-2, as evidenced by the failure of proliferation of adoptively transferred IL-2 receptor alpha- and beta-chain-deficient naive CD8^+^ T cells. Consistent with this, *Jak3* ^–/–^ mice showed elevated serum levels of IL-2 compared to wild-type mice, and interestingly, IL-2 production was due to high levels of accumulation of activated CD4^+^ T cells in *Jak3* ^–/–^ mice along with defective CD4^+^ T regulatory cells. Collectively, these findings reveal previously unidentified unique immune contexts of *Jak3* ^–/–^ mice that cause robust IL-2-driven T cell expansion and have a clinical implication for designing a treatment strategy for human patients with loss-of-function genetic mutations of *Jak3*.

## Introduction

Janus kinase 3 (Jak3) is a member of the Janus family of protein tyrosine kinases and is specifically associated with a cytoplasmic domain of the common receptor gamma-chain (γ_c_) shared by cytokines such as IL-2, IL-4, IL-7, IL-9, IL-15, and IL-21 (1, 2). When these cytokines bind to their specific receptors containing γ_c_, Jak3 is activated and initiates a cascade of signal transduction through the phosphorylation of signal transducer and activator of transcription (STAT) proteins to induce the expression of various genes and subsequent biological functions (3–5). Based on these roles of γ_c_ cytokines, Jak3-deficient mice have several abnormalities similar to those observed in mice lacking γ_c_ cytokines and their corresponding receptors (6–8). In particular, defects of T and B cells and NK cells typically observed in mice lacking Jak3 can be largely attributed to failures in inducing IL-7 and IL-15 signaling, respectively (9–11). Likewise, defects in the development of CD4^+^ T regulatory cells (Tregs) are also apparent in Jak3-deficient mice owing to the inability to induce IL-2 signaling that is involved in the development, maintenance, and function of Tregs (12).

Although all these aforementioned alterations are adequately documented in both mice and humans lacking functional Jak3, the human cases are in general more clearly associated with severe combined immunodeficiency (13–15). Thus, Jak3-deficient mice, unlike patients with loss-of-function mutations of *Jak3* (16), are not completely lymphopenic for T cells in particular, but their numbers (for CD4^+^ but not CD8^+^ T cells) are almost fully restored over time with age (9, 10, 17). Moreover, the T cells restored in Jak3-deficient mice have been shown to display an activated T cell phenotype, such as high and low levels of CD44 and CD62L, respectively (9). Although these large numbers of activated T cells are likely to be associated with a defect in Treg-mediated immunosuppression in Jak3-deficient mice (12, 18), whether these cells indeed contribute to shaping altered immune contexts in these mice remains to be addressed.

In this study, we focused on this issue by investigating proliferative responses of naive CD4^+^ and CD8^+^ T cells adoptively transferred into Jak3-deficient mice. We demonstrated that these mice have a unique IL-2-rich immune environment and thus stimulate a fast and robust form of antigen-independent, but IL-2-dependent, T cell proliferative responses. Our findings highlight the important role of Jak3 in restraining the spontaneous activation of CD4^+^ T cells and thus lowering the production of *in vivo* IL-2 below a certain physiological level at which abnormal T cell proliferation is inhibited while Tregs homeostasis is preserved.

## Methods

### Mice

C57BL/6 (B6), B6.PL (Thy1.1), B6.SJL (Ly5.1) mice were purchased from the Jackson Laboratory. Sources of Foxp3-eGFP mice and OT-I, 2C, 2C.*Cd25^−/−^*, 2C.*Cd122^−/−^*, OT-II, and SMARTA TCR Tg mice were previously described (19, 20) and obtained from Pohang University of Science and Technology (POSTECH). *Jak3^−/−^* mice (9) were also obtained from POSTECH, and generated and maintained by crossing with *Jak3^+/−^* mice. *Jak3^+/+^* or *Jak3^+/−^* mice were used as a littermate control. Unless it is described, 8–10 weeks old mice were used for the experiments according to the protocols approved by the Institutional Animal Care and Use Committee of the Chonnam National University.

### T Cell Purification

Pooled (inguinal, axillary, cervical and mesenteric) lymph node (LN) cells from the indicated mice were prepared for cell sorting as previously described (20). In brief, LN cells were first depleted of non-T cells by using the following biotinylated antibodies; CD11b, CD11c, CD24, CD19, B220, NK1.1 and IMag according to the manufacturer’s protocol (BD Biosciences). Enriched T cells were stained with fluorochrome conjugated antibodies to CD8α, CD4, CD25, CD44, and CD62L for obtaining either CD4^+^ CD25^−^ CD44^lo^ CD62L^hi^ (naive CD4^+^) or CD8α^+^ CD44^lo^ CD62L^hi^ (naive CD8^+^), or in some experiment Foxp3-eGFP^+^ CD4^+^ (CD4^+^ Tregs), and then sorted by using a FACS AriaII (BD Biosciences) or Moflo XDP (Beckman Coulter, Brea, CA, USA) to >95% purity.

### Adoptive Transfer

After purification, T cells were labeled with 5 µM of CFSE (Invitrogen) as previously described (20) and injected intravenously (i.v.) into the indicated hosts. For inducing lymphopenia, the indicated mice were treated with 700 rad of whole-body irradiation (1 day before adoptive transfer).

### Flow Cytometry Analysis

Single-cell suspensions were prepared from lymph nodes and spleens, and were pressed and filtered through cell strainers. For surface staining, isolated cells were stained with the following fluorochrome-conjugated mAbs from Biolegend, eBioscience, or TONBO: CD3 (145-2C11), CD4 (GK1.5 and RM4–5), CD8α (53-6.7), CD25 (PC61.5), CD44 (IM7), CD45.1 (A20), CD45.2 (104), CD62L (MEL-14), CD90.1 (HIS51 or OX-7), and 2C TCR clonotype (1B2) (19). Propidium iodide (PI) (Sigma Aldrich) was used at 500 ng/ml of final concentration for staining of 1–5 × 10^6^ of cells to label dead cells. Flow cytometry samples were run using a LSRII or FACSCanto II (BD Biosciences) and analyzed by FlowJo software (Tree Star).

### Administration of Antibodies *In Vivo*

For the *in vivo* CD4^+^ T cell depletion experiment, 100 µg of anti-CD4 mAb (GK1.5) was injected intraperitoneally (i.p.) four times every 2 days for 7 days before adoptive cell transfer into *Jak3^−/−^* hosts.

### Cytokine ELISA

For detection of *in vivo* IL-2, sera from the indicated mice were collected and analyzed by a standard protocol using a cytokine sandwich ELISA kit for IL-2 (BD Biosciences) according to the manufacturer’s instructions.

### Direct Intracellular Cytokine Staining for *In Vivo* IL-2 Production

As previously described (21), 250 μg brefeldin A (Sigma-Aldrich) was injected i.v. into *Jak3^+/+^* and *Jak3^−/−^* mice. Six hours later, mice were sacrificed, and single-cell suspensions were prepared from spleens on ice in the presence of 10 μg/ml brefeldin A. Splenocytes were immediately Fc-blocked (anti-CD16/32; BD Biosciences) without any exogenous stimulus, surface stained with CD4, CD8α, CD44, and CD62L, fixed and permeabilized with CytoFix/CytoPerm (BD Biosciences), and stained for intracellular cytokine IL-2 (JES6-5H4; BD Biosciences) for flow cytometry.

### Real-Time (RT) PCR

1–2 × 10^6^ spleen cells or FACS-purified CD4^+^ T cells from the indicated mice were used for RNA extraction with NucleoZOL (Macherey-Nagel) and stored at -80 °C before the further steps. Isolation of mRNA was done according to the manufacturer’s instructions. cDNA was synthesized with the M-MLV reverse transcriptase and oligo dT (TAKARA). Real-time RT-PCR was performed with the TaqMan Gene Expression Master Mix using StepOnePlus Real-Time PCR System with TaqMan probe for *Il2* mRNA (Mm00434256_m1; Applied Biosystems).

### Statistical Analysis

An unpaired two-tailed Student’s *t*-test was performed to test statistical significance using Prism (GraphPad Software). Differences in mean values were considered statistically significant at a *P* value of less than 0.05.

## Results

### Robust Proliferative Responses of Naive T Cells in Jak3^–/–^ Hosts

**Figure 1:**
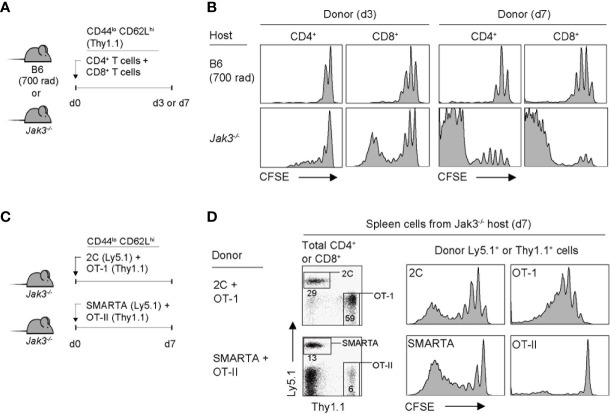
Robust proliferation of naive T cells adoptively transferred into *Jak3^−/−^* mice. **(A)** Schematic diagram for adoptive transfer experiments. A mixture of CFSE-labeled naive CD4^+^ and CD8^+^ T cells purified from B6 mice (Thy1.1) was co-injected i.v. into either irradiated (700 rad) B6 mice or unmanipulated *Jak3^−/−^* mice (1 × 10^6^ cells for each donor per mouse; *n* = 3–5 mice). **(B)** Spleen cells of the recipient mice were analyzed on days 3 and 7 for CFSE dilution by flow cytometry. **(C)** Schematic diagram for adoptive transfer experiments. A mixture of FACS-purified CFSE-labeled either naive 2C (Ly5.1) and OT-I (Thy1.1) CD8^+^ or naive OT-II (Thy1.1) and SMARTA (Ly5.1) CD4^+^ T cells was co-injected i.v. into *Jak3^−/−^* mice (0.5–1 × 10^6^ cells for each donor per mouse; *n* = 2–3 mice). **(D)** Spleen cells of the recipient mice were analyzed on day 7 for CFSE dilution by flow cytometry.

Given the functional association of Jak3 for mediating the signal transduction of γ_c_ cytokines, it has been previously demonstrated that mice lacking Jak3 (or its binding receptor CD132) have a defect in T cell development, especially the CD8^+^ T cell population, due to IL-7 signaling failure (9, 10, 17). To further examine the altered immune contexts of these mice, we adoptively transferred FACS-purified, CFSE-labeled C57BL/6 (B6) naive CD4^+^ or CD8^+^ T cells into *Jak3* ^–/–^ mice (**Figure 1A**). Notably, the donor CD4^+^ and CD8^+^ T cells showed marked proliferation from day 3 and a more potent response on day 7, as evidenced by CFSE dye dilution (**Figure 1B**, bottom, and **Supplementary Figure 1A**). As a control, when compared to > 6 divisions in *Jak3* ^–/–^ mice, the donor cells adoptively transferred into irradiated B6 mice (700 rad) showed only 2–3 rounds of cell divisions (**Figure 1B**, top, and **Supplementary Figure 1A**). These data suggest that the tempo and type of proliferation of T cells observed in *Jak3* ^–/–^ mice are distinctly different from the typically slow rate of lymphopenia-induced homeostatic proliferation (LIP) (22).

To examine whether the aforementioned fast and robust proliferative responses are dependent on abnormal stimulation by undefined antigens that may be present in *Jak3* ^–/–^ hosts, we performed the adoptive transfer experiment using monoclonal T cell receptor (TCR) transgenic CD4^+^ or CD8^+^ T cells. *Jak3* ^–/–^ mice were co-transferred with a mixture of either OT-II and SMARTA CD4^+^ T cells (specific for I-A^b^-restricted ovalbumin 323–339 and lymphocyte choriomeningitis virus glycoprotein 61-80 peptide, respectively) or 2C and OT-I CD8^+^ T cells (specific for H-2K^b^-restricted SIYRYYGL peptide and ovalbumin 257–264 peptide, respectively) (**Figure 1C**). All donor cells showed proliferation, although to varying extents depending on their intrinsic self-reactivity—known to be correlated with expression levels of CD5, a negative regulator of TCR signaling, i.e., SMARTA > OT-II, and OT-I > 2C (23–25)—with SMARTA and OT-I donors demonstrating a greater proliferation than OT-II and 2C donors, respectively (**Figure 1D** and **Supplementary Figure 1B**). These findings suggest that the strong T cell proliferative response observed in *Jak3* ^–/–^ hosts is antigen-independent but dependent on tonic TCR contacts with self-ligands.

### Role of γc Cytokines in Naive T Cell Expansion in *Jak3* ^–/–^ Hosts

**Figure 2:**
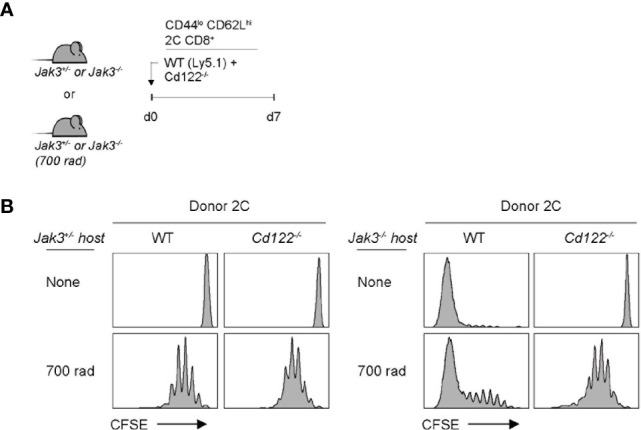
Role of IL-2/IL-15Rβ expression for inducing donor T cell expansion in *Jak3^−/−^* hosts. **(A)** Schematic diagram for adoptive transfer experiments. A mixture of FACS-purified CFSE-labeled naive WT (Ly5.1) and CD122(IL-2/IL-15Rβ)-deficient 2C CD8^+^ T cells was co-injected i.v. into either irradiated (700 rad) or unmanipulated *Jak3^−/−^* and as a control *Jak3^+/−^* mice (0.5 × 10^6^ cells for each donor per mouse; *n* = 2–3 mice). **(B)** Spleen cells of the recipient mice were analyzed on day 7 for CFSE dilution by flow cytometry.

Given the antigen-independent strong T cell proliferation in *Jak3* ^–/–^ hosts, we next examined the involvement of certain γ_c_ cytokines, especially IL-2 and IL-15, both of which have been shown to induce antigen-independent robust T cell proliferation (19). To this end, a mixture of FACS-purified, CFSE-labeled *Cd122* ^–/–^ (IL-2 receptor beta-chain shared for IL-2 and IL-15) and wild-type (WT) naive 2C CD8^+^ T cells was co-transferred into *Jak3* ^–/–^ or control *Jak3^+/^* ^–^ hosts (**Figure 2A**). At day 7 after transfer, in control *Jak3^+/^* ^–^ hosts, both WT and *Cd122* ^–/–^ 2C donor cells remained undivided (**Figure 2B**, top left two). However, in *Jak3* ^–/–^ hosts, while WT 2C donor cells showed strong proliferative responses, *Cd122* ^–/–^ 2C donor cells failed to proliferate (**Figure 2B**, top right two, and **Supplementary Figure 2**). When this adoptive transfer experiment was repeated in the same but irradiated (700 rad) hosts, both WT and *Cd122* ^–/–^ 2C donors showed similar degrees of slow LIP in control *Jak3^+/^* ^–^ hosts (**Figure 2B**, bottom left two), which is known to be IL-7 (and self)-dependent (26–29). However, in irradiated *Jak3* ^–/–^ hosts, WT 2C donor cells showed markedly faster and greater proliferative responses than *Cd122* ^–/–^ 2C donor cells showing only slow rate of LIP (**Figure 2B**, bottom right two, and **Supplementary Figure 2**), suggesting a role of CD122-dependent cytokines other than IL-7.

**Figure 3:**
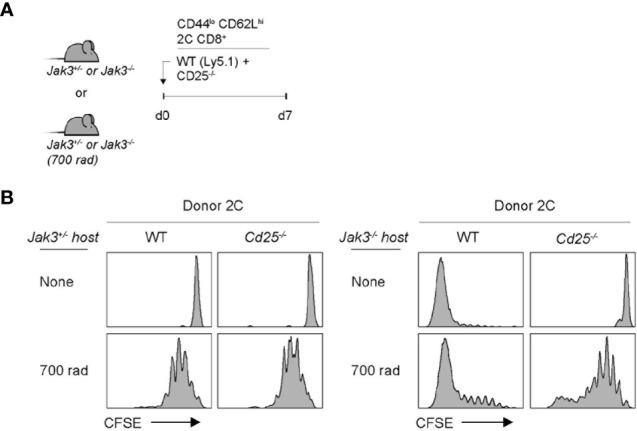
Role of IL-2Rα expression for inducing donor T cell expansion in *Jak3^−/−^* hosts. **(A)** Schematic diagram for adoptive transfer experiments. A mixture of FACS-purified CFSE-labeled naive WT (Ly5.1) and CD25(IL-2Rα)-deficient 2C CD8^+^ T cells was co-injected i.v. into either irradiated (700 rad) or unmanipulated *Jak3^−/−^* and as a control *Jak3^+/−^* mice (0.5 × 10^6^ cells for each donor per mouse; *n* = 2–3 mice). **(B)** Spleen cells of the recipient mice were analyzed on day 7 for CFSE dilution by flow cytometry.

The implication from the above data with *Cd122* ^–/–^ cells as a donor is that IL-2 and/or IL-15 are crucial requirements for driving strong T cell proliferation in *Jak3* ^–/–^ hosts, as the CD122 is a receptor subunit shared for both IL-2 and IL-15. To further examine whether both cytokines are equally required, we performed the same aforementioned adoptive transfer experiments with CD25 (IL-2 receptor alpha-chain)-deficient 2C cells as the donor. For this purpose, a mixture of FACS-purified, CFSE-labeled *Cd25* ^–/–^ and WT naive 2C CD8^+^ T cells was co-transferred into *Jak3* ^–/–^ or control *Jak3^+/^* ^–^ hosts (**Figure 3A**). WT 2C donor cells showed robust proliferation at day 7 in *Jak3* ^–/–^ hosts; however, *Cd25* ^–/–^ 2C donor cells remained undivided (**Figure 3B**, top right two, and **Supplementary Figure 3**). Similarly, for experiments with the same but irradiated *Jak3* ^–/–^ hosts, WT 2C donor cells showed markedly faster and more robust proliferation than *Cd25* ^–/–^ 2C counterparts (**Figure 3B**, bottom right two, and **Supplementary Figure 3**). The latter *Cd25* ^–/–^ 2C cells showed only slow rate of LIP comparable to that of both WT and *Cd25* ^–/–^ 2C donor cells in irradiated *Jak3^+/^* ^–^ hosts, indicating similar responses of these cells to IL-7 under this lymphopenic condition (**Figure 3B**, bottom left two). Together, these findings strongly suggest that the antigen-independent robust proliferation of naive CD8^+^ T cells in *Jak3* ^–/–^ hosts is heavily dependent on the direct engagement of both receptors CD25 and CD122 that are specific for only IL-2.

### Role of CD4^+^ T Cells in High IL-2 Production in *Jak3* ^–/–^ Hosts

**Figure 4:**
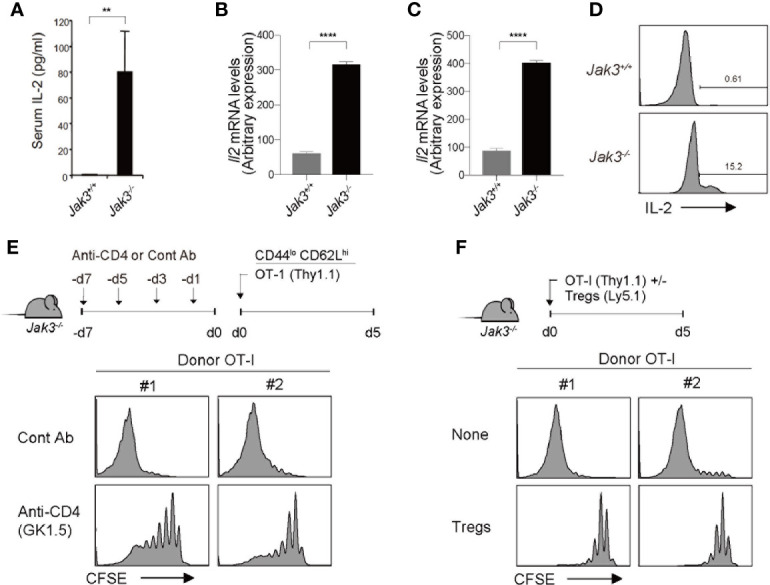
Effect of CD4^+^ T cells on high levels of *in vivo* IL-2 in *Jak3^–/–^* mice. **(A)** *In vivo* levels of IL-2 measured by ELISA with sera from *Jak3^−/−^* mice and as littermate control *Jak3^+/+^* mice (the mean ± SD; *n* = 3-5 mice per group). **(B, C)** Spleen cells **(B)** and FACS-purified CD4^+^ T cells **(C)** from *Jak3^−/−^* and *Jak3^+/+^* mice were analyzed for *Il2* mRNA by quantitative RT-PCR. **(D)** Detection of *in vivo* IL-2 production from *Jak3^−/−^* or *Jak3^+/+^* CD4^+^ T cells by using direct intracellular cytokine staining. **(E)** *Jak3^−/−^* mice were injected i.p. with either anti-CD4 mAb (GK1.5) or as a control isotype IgG (total 4 injections every 2 days; 100 μg per mouse; *n* = 2–4 mice). The mice were injected i.v. with FACS-purified CFSE-labeled naive OT-I CD8^+^ T cells (Thy1.1; 1 × 10^6^ cells per mouse; *n* = 2-4 mice). At day 5 after adoptive transfer, spleen cells of the recipient mice were analyzed for CFSE dilution by flow cytometry. **(F)** FACS-purified CFSE-labeled naive OT-I CD8^+^ T cells (Thy1.1; 1 × 10^6^ cells per mouse) were injected i.v. with or without CD4^+^ Tregs (~0.2 × 10^6^ cells per mouse) purified from Foxp3-GFP mice into *Jak3^−/−^* mice (*n* = 2–4 mice). Spleen cells of the recipient mice were analyzed on day 5 for CFSE dilution by flow cytometry. **P<0.01, ****P<0.0001.

Based on the aforementioned strict dependency on IL-2, we tested the *in vivo* levels of IL-2 in *Jak3* ^–/–^ mice. For this purpose, serum samples were collected from WT and *Jak3* ^–/–^ mice, which were then analyzed for IL-2 detection using ELISA. As shown in **Figure 4A**, *Jak3* ^–/–^ mice showed high serum levels of IL-2 compared to their WT counterparts. Consistent with this finding, *Il2* mRNA expression levels were also markedly more enhanced in *Jak3* ^–/–^ spleen cells than in their WT counterparts (**Figure 4B**). *Il15* mRNA levels were also tested and were no increase (but rather decreased) in *Jak3* ^–/–^ cells compared to WT counterparts (**Supplementary Figure 4A**), suggesting a negligible role of IL-15.

The direct requirement of IL-2 was also confirmed by an *in vivo* blocking experiment, in which *Jak3* ^–/–^ hosts were administered with a monoclonal antibody to either IL-2 or IL-2 receptor beta-chain (clones JES6.1 and TM-β1, respectively; **Supplementary Figure 4B**); the results clearly showed that the JES6.1 and TM-β1 injection (relative to PBS control) are both suppressive to a similar extent for the expansion of OT-I donor cells adoptively transferred into *Jak3* ^–/–^ hosts. These data, together with the aforementioned *in vivo* data with 2C donor cells lacking IL-2 receptors (i.e., CD25 and CD122) (**Figures 2**, **3**), further support our conclusion that strong T cell proliferative responses are driven exclusively by higher concentrations of *in vivo* IL-2 present in *Jak3* ^–/–^ mice.

Given the rich supply of *in vivo* IL-2 in *Jak3* ^–/–^ mice, our prior data that, despite similar degree of cell divisions, WT 2C cells exhibited ~3-5 times lower expansion in irradiated *Jak3* ^–/–^ hosts than in non-irradiated counterparts (**Figures 2**, **3** and **Supplementary Figures 2, 3**) suggest a radiosensitive nature of IL-2-producing cells. With respect to the radiosensitive source of producing such high levels of IL-2 *in vivo*, the role of *Jak3* ^–/–^ CD4^+^ T cells was considered of particular importance, as these cells exhibited highly activated phenotypes (i.e., CD44^hi^CD62L^lo^) and were shown to gradually accumulate in sufficient numbers—comparable or even higher than those of WT controls—in *Jak3* ^–/–^ mice over time with age (9). In support of this, *Il2* mRNA expression levels and *in vivo* IL-2 productions were higher in *Jak3* ^–/–^ CD4^+^ T cells than in their WT counterparts (**Figures 4C, D**). To further confirm the possible role of *Jak3* ^–/–^ CD4^+^ T cells as a major source of high levels of *in vivo* IL-2, *Jak3* ^–/–^ mice were intraperitoneally administered a monoclonal antibody against CD4 for depleting host CD4^+^ T cell populations (clone GK1.5) for 7 days prior to starting adoptive transfer experiments (**Figure 4E**, top). The antibody-treated *Jak3* ^–/–^ mice were adoptively transferred with FACS-purified, CFSE-labeled OT-I naive CD8^+^ T cells, and 5 days later, the proliferation of these cells was analyzed. Proliferation of OT-I donor cells was substantially reduced in GK1.5 mAb-treated *Jak3* ^–/–^ hosts compared to control Ab-treated *Jak3* ^–/–^ hosts (**Figure 4E**, bottom, and **Supplementary Figure 4C**), suggesting a role for *Jak3* ^–/–^ CD4^+^ T cells as an *in vivo* IL-2 resource.

Based on this role of *in vivo* IL-2, which is produced by activated CD4^+^ T cells in *Jak3* ^–/–^ mice, we tested the effect of CD4^+^ Tregs, as this inhibitory CD4^+^ population is absent in *Jak3* ^–/–^ mice (12, 18). To this end, FACS-purified, CFSE-labeled OT-I naive CD8^+^ T cells were adoptively transferred with or without FACS-purified CD4^+^ Tregs (from Foxp3-GFP mice) into *Jak3* ^–/–^ hosts (**Figure 4F**, top). Proliferative responses of OT-I donor cells were markedly inhibited by the co-injection of Tregs compared to control non-injected *Jak3* ^–/–^ hosts (**Figure 4F**, bottom, and **Supplementary Figure 4D**), and this finding was consistent with the prior notion that high level of CD25 expression on Tregs could deprive IL-2 from effector T cells and inhibit their proliferation by predominantly consuming IL-2 *in vivo* (30–32).

Collectively, these data indicate that *Jak3* ^–/–^ mice have a unique immune environment, where large numbers of activated CD4^+^ T cells (and conversely a scarcity of CD4^+^ Tregs) result in abnormally high levels of IL-2 production, which can induce the strong proliferation of adoptively transferred naive T cells in an antigen-independent manner.

## Discussion

*Jak3* ^–/–^ mice have been demonstrated to experience profound immunodeficiency, with developmental defects in various immune cell types, including T cells (both αβ and γδ T cells), B cells, and NK cells (9–11). These defects have been largely attributed to defective downstream signaling of Jak3-associated γ_c_ cytokines, particularly IL-7 and IL-15 (6–8). In addition, defective IL-2 signaling of Jak3-deficient mice leads to Treg deficiency, which is consistent with mice lacking IL-2 or its receptor components (i.e., CD25, CD122, and CD132) (12, 33, 34). Although the latter is associated with the gradual accumulation of activated CD4^+^ T cell populations in *Jak3* ^–/–^ mice (9, 10, 17), its physiological impacts remain unclear. Here, we demonstrated a previously unidentified phenotype of Jak3-deficient mice by showing that *Jak3* ^–/–^ CD4^+^ T cells produced extremely high amounts of IL-2 *in vivo*, and consequently, adoptively transferred naive T cells could undergo a rapid and intense form of antigen-independent proliferation. Notably, this type of proliferation—even though antigen-independent—was still influenced by the intrinsic TCR affinity/avidity for self-ligands. Such self-dependency was in close agreement with previous findings demonstrating strong IL-2-driven T cell proliferation both *in vitro* and *in vivo* (19, 20). Therefore, our findings reveal a unique immune context, i.e., supraphysiological levels of *in vivo* IL-2, of Jak3-deficient mice and further strengthen the prevailing notion that even a single γ_c_ cytokine (IL-2) plays a crucial role in promoting massive bystander expansions of naive T cell populations.

The strong proliferation of adoptively transferred naive CD8^+^ T cells observed in *Jak3* ^–/–^ mice closely resembles those observed in mice lacking CD122 (IL-2 receptor beta, IL-2Rβ) and CD132 (common γ_c_ receptor) in terms of their tempo and robustness as well as strict dependency on two stimuli, namely γ_c_ cytokines and TCR contacts with self-ligands (19, 35). However, the difference was the type of cytokines involved; while IL-2 is a major driver in *Jak3* ^–/–^ mice, mixtures of 2-3 γ_c_ cytokines—either IL-2 and IL-15 or IL-7, IL-2, and IL-15—act as stimulators in *Cd122* ^–/–^ and *Cd132* ^–/–^ mice, respectively. Moreover, the reason for such enhanced levels of these cytokines in *Cd122* ^–/–^ and *Cd132* ^–/–^ mice was largely accounted for by the lack of the receptors involved in specific binding to either IL-2 and IL-15 (for *Cd122* ^–/–^ mice) or IL-7, IL-2, and IL-15 (for *Cd132* ^–/–^ mice), thereby leading to *in vivo* accumulation of cytokines that would otherwise be consumed (19, 35). In this regard, high levels of *in vivo* IL-2 in *Jak3* ^–/–^ mice was odd at first glance, since these mice have a defect not in the IL-2 receptors but in the downstream signaling. However, based on our data showing the reduced T cell expansion caused by CD4^+^ T cell depletion (using GK1.5 mAb) and co-transfer with Tregs, we propose that CD4^+^ T cells in *Jak3* ^–/–^ mice are overtly activated—presumably by their uncontrolled reactivity to self-antigens—due to the lack of Tregs, which in turn produces IL-2 markedly beyond a physiological level that is sufficient for stimulating intense proliferative responses of adoptively transferred naive CD8^+^ T cells. Whether prior Tregs reconstitution can restore abnormal immune contexts (i.e., high levels of *in vivo* IL-2) of Jak3-deficient mice will thus be interesting to address in future studies.

Despite a few cases of *Jak3* mutations in humans, whether the phenomenon observed in *Jak3* ^–/–^ mice is similarly reproduced in clinical patients lacking functional Jak3 remains elusive. In this regard, clinical cases, albeit a few, showing that activated/memory T cells have been associated with *Jak3* mutations support this possibility (36, 37). Nevertheless, the majority of patients with defective Jak3 are associated with severe combined immunodeficiency and thus compounding effects due to increased susceptibility to infection cannot be easily excluded (13–16, 38). In summary, our results further extend our understanding of the physiological impact of Jak3 deficiency, which can cause the uncontrolled activation and accumulation of self-reactive CD4^+^ T cells and accordingly abnormally high levels of *in vivo* IL-2 production. These findings also have an implication for rationale treatment strategies for patients with a loss-of-function *Jak3* mutation—especially those concerning graft-versus-host reaction from transplacentally acquired maternal or donor allogeneic T cells after bone marrow transfusions.

## Data Availability Statement

The raw data supporting the conclusions of this article will be made available by the authors, without undue reservation.

## Ethics Statement

The animal study was reviewed and approved by Animal Experimental and Ethic Committee of the Chonnam National University.

## Author Contributions

G-WL performed all major experiments. G-WL and J-HC designed experiments and analyzed and interpreted the data. S-WL, JK, and Y-JJ helped some experiments, and C-HY and H-OK contributed to this study with valuable discussion and comments. G-WL and J-HC wrote the manuscript. All authors contributed to the article and approved the submitted version.

## Funding

This work was supported by National Research Foundation funded by the Korean Ministry of Science and ICT (2018R1A5A2024181 and 2020M3A9G3080281 for J-HC). 

## Conflict of Interest

The authors declare that the research was conducted in the absence of any commercial or financial relationships that could be construed as a potential conflict of interest.
