# Supplementary material for: Supraphysiological Levels of IL-2 in Jak3-Deficient Mice Promote Strong Proliferative Responses of Adoptively Transferred Naive CD8+ T Cells
Source: Front Immunol. 2021 Jan 28;11:616898. doi: 10.3389/fimmu.2020.616898 (PMC7876067; doi:10.3389/fimmu.2020.616898)
Supplement: Supplementary file 1 [file DataSheet_1.docx]

Supplementary Material

**Supraphysiological levels of IL-2 in Jak3-deficient mice promote strong proliferative responses of adoptively transferred naive CD8^+^ T cells**

Gil-Woo Lee^1,2,3^, Sung-Woo Lee^1,2,3^, Juhee Kim^1^, Young-Jun Ju^4^, Hee-Ok Kim^3^, Cheol-Heui Yun^4^, and Jae-Ho Cho^2,3,*^

^1^Division of Integrative Biosciences and Biotechnology, Pohang University of Science and Technology, Pohang, South Korea.

^2^Medical Research Center for Combinatorial Tumor Immunotherapy, Department of Microbiology and Immunology, Chonnam National University Medical School, Jeonnam, South Korea.

^3^Immunotherapy Innovation Center, Chonnam National University Medical School, Hwasun Hospital, Jeonnam, South Korea.

^4^Department of Agricultural Biotechnology and Research Institute of Agriculture and Life Sciences, Seoul National University, Seoul, South Korea.

**Correspondence:** Jae-Ho Cho**:** jh_cho@chonnam.ac.kr

**Supplementary Figures**

**Supplementary Figure 1**

**
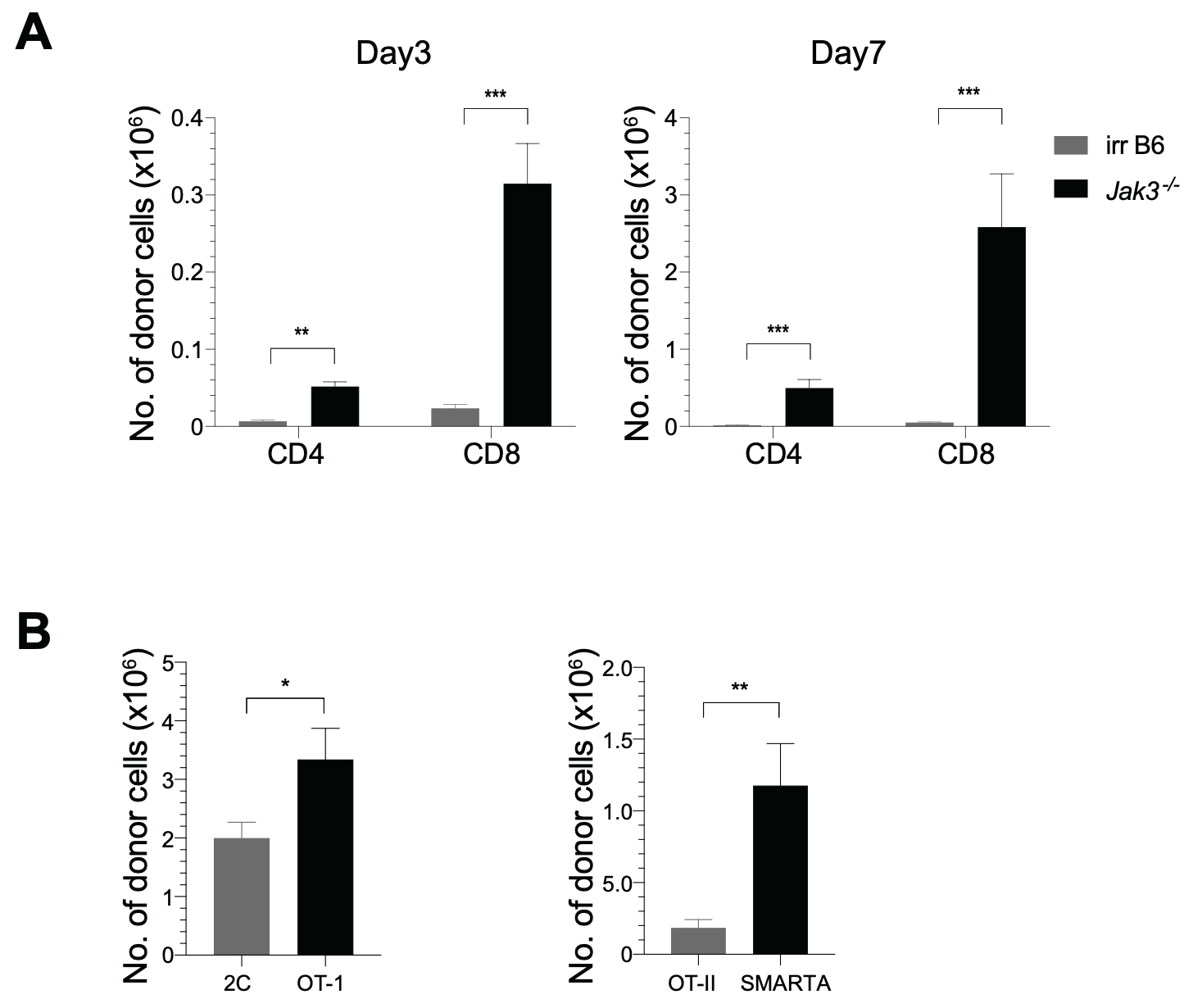
**

**Supplementary Figure 1**. **Robust proliferation of naive T cells adoptively transferred into *Jak3^−/−^* mice.** **(A)** A mixture of CFSE-labeled naive CD4^+^ and CD8^+^ T cells purified from B6 mice (Thy1.1) was co-injected i.v. into either irradiated (700 rad) B6 mice or unmanipulated *Jak3^−/−^* mice (1 × 10^6^ cells for each donor per mouse; *n* = 3-5 mice). Spleen cells of the recipient mice were analyzed on days 3 (left) and 7 (right) by flow cytometry for total donor cell recovery. Data shown are the mean ± SD (*n* = 3-5 mice per group). **(B)** A mixture of FACS-purified CFSE-labeled either naive 2C (Ly5.1) and OT-I (Thy1.1) CD8^+^ or naive OT-II (Thy1.1) and SMARTA (Ly5.1) CD4^+^ T cells was co-injected i.v. into *Jak3^−/−^* mice (0.5-1 × 10^6^ cells for each donor per mouse; *n* = 2-3 mice). Spleen cells of the recipient mice were analyzed on day 7 by flow cytometry for total donor cell recovery. Data shown are the mean ± SD (*n* = 2-3 mice per group). * *P < 0.05*, ** *P < 0.01*, *** *P < 0.001.*

**Supplementary Figure 2**


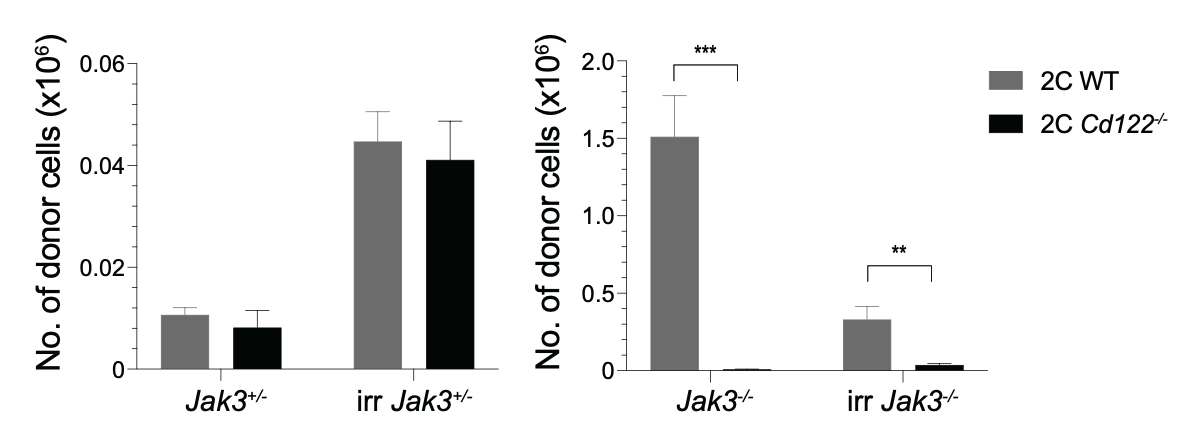


**Supplementary Figure 2**. **Role of IL-2/IL-15Rβ expression for inducing donor T cell expansion in *Jak3^−/−^* hosts.** A mixture of FACS-purified CFSE-labeled naive WT (Ly5.1) and CD122(IL-2/IL-15Rβ)-deficient 2C CD8^+^ T cells was co-injected i.v. into either irradiated (700 rad) or unmanipulated *Jak3^−/−^* and as a control *Jak3^+/−^* mice (0.5 × 10^6^ cells for each donor per mouse; *n* = 2-3 mice). Spleen cells of the recipient mice were analyzed on day 7 by flow cytometry for total donor cell recovery. Data shown are the mean ± SD (*n* = 2-3 mice per group). ** *P < 0.01*, *** *P < 0.001*.

**Supplementary Figure 3**


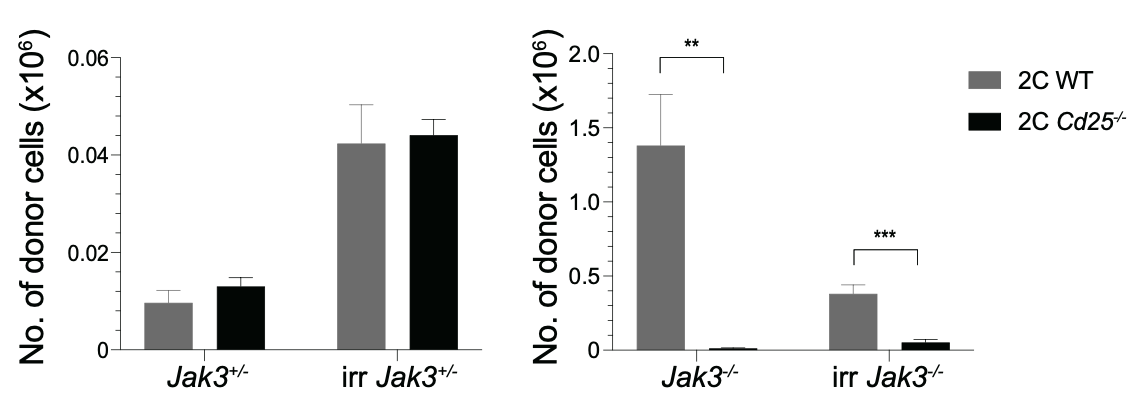


**Supplementary Figure 3**. **Role of IL-2Rα expression for inducing donor T cell expansion in *Jak3^−/−^* hosts.** Schematic diagram for adoptive transfer experiments. A mixture of FACS-purified CFSE-labeled naive WT (Ly5.1) and CD25(IL-2Rα)-deficient 2C CD8^+^ T cells was co-injected i.v. into either irradiated (700 rad) or unmanipulated *Jak3^−/−^* and as a control *Jak3^+/−^* mice (0.5 × 10^6^ cells for each donor per mouse; *n* = 2-3 mice). Spleen cells of the recipient mice were analyzed on day 7 by flow cytometry for total donor cell recovery. Data shown are the mean ± SD (*n* = 2-3 mice per group). ** *P < 0.01*, *** *P < 0.001*.

**Supplementary Figure 4**

**
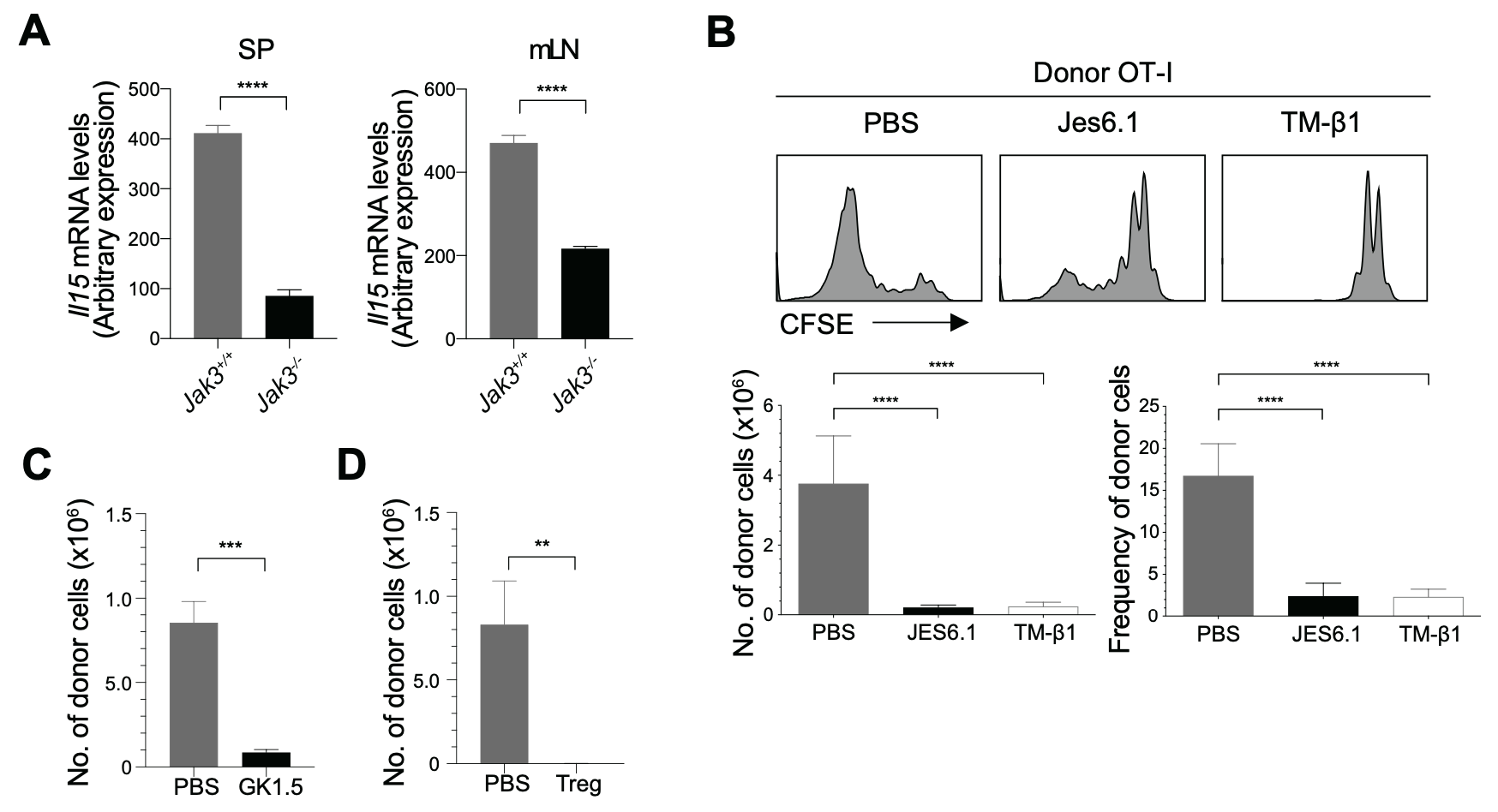
**

**Supplementary Figure 4**. **Effect of CD4^+^ T cells on high levels of *in vivo* IL-2 in *Jak3^−/−^* mice. (A)** Spleen and mesenteric lymph node (mLN) cells from *Jak3^−/−^* and *Jak3^+/+^* mice were analyzed for *Il15* mRNA by quantitative RT-PCR (the mean ± SD; *n* = 3-5 mice per group). **(B)** *Jak3^−/−^* mice were injected i.p. with either anti-IL-2 mAb (JES6.1) or anti-IL-2Rβ mAb (TM-β1) (on days -3 and 0; 100 μg per mouse; *n* = 2-3 mice). The mice were then injected i.v. with FACS-purified CFSE-labeled naive OT-I CD8^+^ T cells (Thy1.1; 1 × 10^6^ cells per mouse; *n* = 2-3 mice). At day 7 after adoptive transfer, spleen cells of the recipient mice were analyzed by flow cytometry for CFSE dilution and donor cell recovery (the mean ± SD; *n* = 2-3 mice per group). **(C)** *Jak3^−/−^* mice were injected i.p. with either anti-CD4 mAb (GK1.5) or as a control isotype IgG (total 4 injections every 2 days; 100 μg per mouse; *n* = 2-4 mice). The mice were injected i.v. with FACS-purified CFSE-labeled naive OT-I CD8^+^ T cells (Thy1.1; 1 × 10^6^ cells per mouse; *n* = 2-4 mice). At day 5 after adoptive transfer, spleen cells of the recipient mice were analyzed by flow cytometry for donor cell recovery (the mean ± SD; *n* = 2-4 mice per group). **(D)** FACS-purified CFSE-labeled naive OT-I CD8^+^ T cells (Thy1.1; 1 × 10^6^ cells per mouse) were injected i.v. with or without CD4^+^ Tregs (~0.2 × 10^6^ cells per mouse) purified from Foxp3-GFP mice into *Jak3^−/−^* mice (*n* = 2-4 mice). Spleen cells of the recipient mice were analyzed on day 5 by flow cytometry for donor cell recovery (the mean ± SD; *n* = 2-4 mice per group). ** *P<0.01*, *** *P < 0.001*, **** *P<0.0001.*
